# Adaptive Square-Root Unscented Particle Filtering Algorithm for Dynamic Navigation

**DOI:** 10.3390/s18072337

**Published:** 2018-07-18

**Authors:** Wenhui Wei, Shesheng Gao, Yongmin Zhong, Chengfan Gu, Gaoge Hu

**Affiliations:** 1 School of Geological Engineering and Surveying and Mapping, Chang’An University, Xi’an 710064, China; 2 School of Automatics, Northwestern Polytechnical University, Xi’an 710072, China; gshshnpu@nwpu.edu.cn (S.G.); hugaoge1111@126.com (G.H.); 3 School of Engineering, RMIT University, Bundoora, Melbourne 3083, Australia; yongmin.zhong@rmit.edu.au; 4 Department of Mechanical Engineering, University of Melbourne, Parkville, Melbourne 3010, Australia; chengfan.gu@gmail.com

**Keywords:** performance analysis, particle filter, adaptive filtering, Cholesky factorization, integrated navigation

## Abstract

This paper presents a new adaptive square-root unscented particle filtering algorithm by combining the adaptive filtering and square-root filtering into the unscented particle filter to inhibit the disturbance of kinematic model noise and the instability of filtering data in the process of nonlinear filtering. To prevent particles from degeneracy, the proposed algorithm adaptively adjusts the adaptive factor, which is constructed from predicted residuals, to refrain from the disturbance of abnormal observation and the kinematic model noise. Cholesky factorization is also applied to suppress the negative definiteness of the covariance matrices of the predicted state vector and observation vector. Experiments and comparison analysis were conducted to comprehensively evaluate the performance of the proposed algorithm. The results demonstrate that the proposed algorithm exhibits a strong overall performance for integrated navigation systems.

## 1. Introduction

Nonlinear filtering is ubiquitous in many areas such as integrated navigation system, geodetic positioning, automatic control, information fusion and signal processing. It aims to estimate the state of a nonlinear dynamic system from observations. The extended Kalman filtering (EKF) is a widely used filtering method for nonlinear systems [1,2]. It linearizes nonlinear system equations by a truncated Taylor series expansion and then applies the linear Kalman filter to the linearized system equations. However, it still requires the linearized state obey the Gaussian distribution, which is usually not consistent with practical applications [3]. Further, when the probability function of state distribution involves multiple peaks, the filtering solution will be biased or even divergent [4]. EKF also involves a complicated calculation process of solving Jacobian matrix. The unscented Kalman filter (UKF) avoids the linearization error of EKF by approximating the probability density of state distribution using unscented transformation (UT) [5,6]. It does not need to calculate Jacobian matrix. However, this method inherits the linear update structure of the Kalman filtering and also requires the system state obey the Gaussian distribution, which is unsuitable for nonlinear systems with non-Gaussian system state model.

The particle filtering (PF) is an optimal recursive Bayesian filtering method based on Monte Carlo simulation [7,8]. Since it is not limited by system linearity and the system state is not subject to the Gaussian distribution, this method can deal with nonlinear system models with non-Gaussian system state [8,9,10]. However, PF suffers from the particle degeneracy phenomenon and the accuracy largely depends on the choice of importance sampling density function and resampling scheme [11,12,13]. Research efforts have been focused on design of a good importance sampling density function and improvement of the resampling scheme to improve the PF performance [14,15,16,17]. The unscented particle filtering (UPF) is a method to obtain a better importance sampling density function using UT to approximate the posterior probability density function of the state [17,18,19,20]. However, this method still suffers from the particle degeneracy phenomenon if the dynamic system is affected by the disturbances of abnormal observation and kinematic model noise [10,17,20]. In fact, the disturbances caused by abnormal observation or kinematic model noise are unavoidable in practical engineering applications [21,22]. In addition, due to the use of a large number of particles, PF also causes an expensive computational load. The parallel implementation within a shared-memory architecture [23], reduced-order system modelling to reduce the filtering dimensionality [24] and improvement of the algorithm structure can be used to improve the computational performance of PF [18,20].

The robust adaptive filtering is a method to handle the problem of degradation or divergence due to abnormal observation and kinematic model noise. It robustly estimates the covariance matrix of observation noise and adaptively adjusts the covariance matrix of state noise by augmenting the adaptive factor into the covariance matrix of state prediction to improve the filtering robustness [21,22,25]. Yang et al. reported a robust adaptive filter by combining the robust maximum-likelihood estimation with the adaptive filtering process to adaptively adjust the weight matrix of predicted parameters according to the difference between system observation and model information [26]. This filter can be adaptively converted into the classical Kalman filter, adaptive Kalman filter and Sage filter by modifying the weight matrix and adaptive factor. Ding et al. reported a process noise scaling method by improving the robustness of adaptive filtering, where the status of the filter operation is monitored using covariance matching [27]. Gao et al. [28,29] combined the random weighting concept with adaptive filtering for a dynamic navigation system. This method establishes unbiased random weighting estimations of observation and state noises and feedbacks them to the kinematic and observation models of a dynamic navigation system to improve the filtering robustness. Azam et al. [30,31] studied the online input estimation techniques to handle cases in which the input of the robust adaptive filtering is unknown.

There are few studies focusing on the use of robust adaptive filtering to improve the UPF performance. Xue et al. [32] reported a new robust adaptive unscented particle filtering algorithm. In order to prevent particles from degeneracy, this algorithm adaptively determines the equivalent weight function according to robust estimation and adaptively adjusts the adaptive factor constructed from predicted residuals to inhibit the disturbances of abnormal observation and kinematic model noise. However, due to the adaptive adjustment to the covariance matrices of predicted state vector and observation vector, this algorithm cannot guarantee the covariance matrices in the filtering process are positive definite, leading to the illness of the filtering process [33]. The square-root filtering provides a solution to overcome this problem. It can improve the update accuracy of covariance matrices by Cholesky factorization and effectively avoid the negative definiteness of covariance matrices.

This paper presents a new adaptive square-root unscented particle filtering (ASUPF) algorithm by combining adaptive filtering and square-root filtering into UPF. This algorithm uses adaptive factors to reasonably control the statistics of observation and kinematic models to inhibit the disturbances of systematic noises, thus preventing particles from degeneracy. Further, Cholesky factorization is used to suppress the negative definiteness of the covariance matrices of predicted state vector and observation vector. Simulation and experimental analyses as well as comparison analysis with the existing nonlinear filtering algorithms were conducted to comprehensively evaluate the performance of the proposed nonlinear filtering algorithm for dynamic navigation.

## 2. Construction of Adaptive Factor

The role of the adaptive factor in the filtering process is to correct the predicted values using the observation values, as well as to estimate and correct the unknown or inaccurate system model parameters and noise statistics.

Consider the following nonlinear system

(1) $$\begin{array}{l} x_{k}=f(x_{k-1},v_{k-1})\\ y_{k}=h(x_{k},n_{k}) \end{array}$$

where $x_{k}\in R^{n}$ is the state vector at epoch k, $y_{k}\in R^{n}$ is the system observation, $v_{k}\in R^{n}$ is the process noise with the variance $R_{k}$, $n_{k}\in R^{n}$ is the observation noise with the variance $Q_{k}$, both f(⋅) and h(⋅) are a nonlinear function and k=0,1,⋯,N is the sampling epoch.

According to the theory of robust estimation, the predicted residual vector reflects the disturbance of the dynamic system, since it contains the state information that has not been corrected by observation. Therefore, the predicted residual vector can be used as the variable to construct the error discriminant statistic and adaptive factor of the kinematic model. The predicted residual vector at time k can be expressed as

(2) $${\bar{V}}_{k}=y_{k}-{\bar{y}}_{k}$$

where ${\bar{y}}_{k}$ is the predicted observation vector.

Accordingly, the error discriminant statistic can be constructed by using ${\bar{V}}_{k}$

(3) $$\Delta {\bar{V}}_{k}={\left(\frac{{\left({\bar{V}}_{k}\right)}^{T}{\bar{V}}_{k}}{\mathrm{tr}(P_{y_{k}y_{k}})}\right)}^{\frac{1}{2}}$$

where $\Delta {\bar{V}}_{k}$ is the error of the predicted residual vector, $P_{y_{k}y_{k}}$ is the covariance matrix of the predicted observation vector and tr(⋅) represents the trace of a matrix. According to (3), three kinds of adaptive factor can be constructed, namely the two-segment function adaptive factor, three-segment function adaptive factor and exponential function adaptive factor [26].

The two-segment function adaptive factor can be constructed as

(4) αk={1|ΔV¯k|≤cc|ΔV¯k||ΔV¯k|>c

where $\alpha_{k}$ represents the adaptive factor, satisfying $0\leq \alpha_{k}\leq 1$ and c=1.0~2.5 is a constant.

The three-segment function adaptive factor can be constructed as

(5) $$\alpha_{k}=\;\{\begin{array}{ll} \;1 & \left|\Delta {\bar{V}}_{k}\right|\leq c_{0}\;\\ \frac{c_{0}}{\left|\Delta {\bar{V}}_{k}\right|}{\left(\frac{c_{1}-\left|\Delta {\bar{V}}_{k}\right|}{c_{1}-c_{0}}\right)}^{2} & c_{0}<\left|\Delta {\bar{V}}_{k}\right|\leq c_{1}\\ \;0 & \left|\Delta {\bar{V}}_{k}\right|>c_{1} \end{array}$$

where $\alpha_{k}$ satisfies $0\leq \alpha_{k}\leq 1$, $c_{0}=1.0\sim 1.5$ and $c_{1}=3.0\sim 8.5$ are constants.

The exponential function adaptive factor can be constructed as

(6) $$\alpha_{k}=\left\{ \begin{array}{ll} 1 & \left|\Delta {\bar{V}}_{k}\right|\leq c\\ e^{-{\left(\left|\Delta {\bar{V}}_{k}-c\right|\right)}^{2}}\; & \left|\Delta {\bar{V}}_{k}\right|>c \end{array}\right.$$

where $\alpha_{k}$ satisfies $0\leq \alpha_{k}\leq 1$, c is a constant and its value is usually 1.5.

## 3. Adaptive Square-Root Unscented Particle Filtering Algorithm

Abnormal interference can be caused by various system factors such as the additional thrust change of carrier’s manoeuvre, mechanical disturbance, sensors anomaly and systematic noises and various environmental factors such as air resistance, weather conditions and radiation. It will lead to a sudden increase in observation error (i.e., the observation abnormality), or the inconformity of the navigation kinematic model with the actual model (i.e., the model abnormality), leading to a decrease in the accuracy of dynamic navigation. Combined with the advantages of adaptive filtering and square-root filtering, an ASUPF algorithm for nonlinear systems is proposed in this section. This algorithm selects appropriate adaptive factors to control the information of the kinematic and observation models and suppresses the influence of abnormal interference, to improve the filtering accuracy. Simultaneously, in order to suppress the negative definiteness of the covariance matrices, Cholesky factorization is applied to the filtering process.

Consider the nonlinear system described as (1), the ASUPF algorithm includes the following steps.

**Step 1:** Initialization

Draw N sampling points according to the initial mean and variance. For k=0, $x_{0}^{i}\sim p(x_{0})$, i=1,2,⋯,N. Assume

(7) $$\begin{array}{c} {\bar{x}}_{0}^{i}=E[x_{0}^{i}]\\ S_{0}^{i}=chol\left\{E[(x_{0}^{i}-{\bar{x}}_{0}^{i}){\left(x_{0}^{i}-{\bar{x}}_{0}^{i}\right)}^{T}]\right\}\\ w_{0}^{i}=p(y_{0}|x_{0}^{i}) \end{array}$$

where $x_{0}^{i}$ and ${\bar{x}}_{0}^{i}$ represent the ith initial particle and its estimated value, $S_{0}^{i}$ represents the ith Cholesky factorization factor at the initial time, $w_{0}^{i}$ denotes the initial weight of the ith particle and chol{⋅} is the Cholesky factorization operator.

**Step 2:** For k=1,2,⋯,N, conduct importance sampling.

(i) Calculate the Sigma points and weights
  (8) $$\left\{ \begin{array}{l} x_{0,k-1}^{i}={\bar{x}}_{k-1}^{i}\\ x_{j,k-1}^{i}={\bar{x}}_{k-1}^{i}+\sqrt{\left(N+\lambda \right)}S_{k-1}^{i}\;j=1,\cdots ,N\\ x_{j,k-1}^{i}={\bar{x}}_{k-1}^{i}-\sqrt{\left(N+\lambda \right)}S_{k-1}^{i}\;j=n+1,\cdots ,2N \end{array}\right.\;$$
  (9) $$\left\{ \begin{array}{l} W_{0}^{m}=\lambda /(N+\lambda )\\ W_{0}^{c}=\lambda /(N+\lambda )+(1-\alpha^{2}+\beta )\\ W_{j}^{m}=W_{j}^{c}=0.5/(N+\lambda )\;\;j=1,\cdots ,2N \end{array}\right.$$
  where $x_{j,k-1}^{i}$ represents the jth Sigma point, $W_{j}$ represents the weight of the jth Sigma point and $\sum W_{j}=1$, j=0,1,⋯,2N. $\lambda =\alpha^{2}(N+\kappa )$ is the size factor, κ is the second-order size factor, N is the number of particles, α is the factor determining the extent of sample distribution with respect to the predicted state mean and $10^{-3}<\alpha \leq 1$. β is usually determined according to the prior knowledge of the distribution of x and β=2 is optimal for the Gaussian distribution.
(ii) Predict and update the particles using UKF

According to the kinematic model, the predicted state vector is expressed as

(10) $$\chi_{j,k/k-1}^{i}=f(\chi_{j,k-1}^{i})\;$$

The estimate of the predicted state vector is calculated by

(11) $${\bar{x}}_{k/k-1}^{i}=\sum_{j=0}^{2N}W_{j}^{m}\chi_{j,k/k-1}^{i}\;$$

Applying Cholesky factorization to the covariance matrix of the predicted state vector yields

(12) $$S_{k/k-1}^{i}=qr\left\{\left[\sqrt{W_{1}^{c}}(x_{1:2n,k/k-1}^{i}-{\bar{x}}_{k/k-1}^{i})\;\sqrt{P_{W_{k}}}\right]\right\}\;$$

(13) $$S_{k/k-1}^{i}=cholupdate\left\{S_{k/k-1}^{i},x_{0,k/k-1}^{i}-{\bar{x}}_{k/k-1}^{i},W_{0}^{c}\right\}\;$$

where cholupdate{⋅} represents the update operator of Cholesky factorization factor.

By using the adaptive factor $\alpha_{k}^{i}$, $S_{k/k-1}^{i}$ can be modified

(14) $${\bar{S}}_{k/k-1}^{i}=S_{k/k-1}^{i}/\sqrt{\alpha_{k}^{i}}\;$$

where $\alpha_{k}^{i}$ is constructed as (4) and the variance $P_{y_{k}y_{k}}^{i}$ of observation information can be calculated by $S_{y_{k}}^{i}$ and $S_{{\hat{y}}_{k}}^{i}$.

According to the observation model, the observation vector can be written as

(15) $$Y_{k|k-1}^{i}=h(\chi_{k|k-1}^{i})\;$$

The estimate of the observation vector is calculated as

(16) $${\bar{y}}_{k/k-1}^{i}=\sum_{j=0}^{2N}W_{j}^{c}Y_{j,k|k-1}^{i}\;$$

Applying Cholesky factorization to the covariance matrix of the observation vector yields

(17) $$S_{y_{k}}^{i}=qr\left\{\left[\sqrt{W_{1}^{c}}(Y_{1:2n,k/k-1}^{i}-{\bar{y}}_{k/k-1}^{i})\;\sqrt{P_{k-1}^{i}}\right]\right\}\;$$

(18) $$S_{{\hat{y}}_{k}}^{i}=cholupdate\left\{S_{{\hat{y}}_{k}}^{i},Y_{0,k/k-1}^{i}-{\bar{y}}_{k/k-1}^{i},W_{0}^{c}\right\}\;$$

where qr{⋅} represents the QR factorization of matrices.

The covariance matrix of $\chi_{j,k|k-1}^{i}$ and $Y_{j,k|k-1}^{i}$ can be obtained as

(19) $$P_{x_{k}y_{k}}=\sum_{j=0}^{2N}W_{j}^{c}[(\chi_{j,k|k-1}^{i}-{\bar{x}}_{k|k-1}^{i}]\cdot {\left[Y_{j,k|k-1}^{i}-{\bar{y}}_{k|k-1}^{i}\right]}^{T}\;$$

Update the state vector

(20) $${\bar{x}}_{k}^{i}={\bar{x}}_{k|k-1}^{i}+K_{k}^{i}(y_{k}-{\bar{y}}_{k|k-1}^{i}).\;$$

Update the covariance matrix of the estimated state vector

(21) $$U^{i}=K_{k}^{i}S_{{\hat{y}}_{k}}^{i}\;$$

(22) $$S_{k}^{i}=cholupdate\left\{{\bar{S}}_{k/k-1}^{i},U^{i},-1\right\}\;$$

(23) $$P_{k}^{i}=S_{k}^{i}{\left(S_{k}^{i}\right)}^{T}\;$$

where the gain matrix is expressed as

(24) $$K_{k}^{i}=(P_{x_{k}y_{k}}^{i}/{\left(S_{y_{k}}^{i}\right)}^{T})/S_{y_{k}}^{i}\;$$

It can be seen from above that the covariance matrix of the state vector is directly transmitted and updated in the form of Cholesky factorization factor, thus ensuring the positive definiteness of the covariance matrix and enhancing the numerical stability of the update process of the covariance matrix. When the kinematic model is disturbed, the predicted residual increases and the adaptive factor $\alpha_{k}^{i}$ decreases, leading to the reduced utilization of the predicted state. Accordingly, the interference of model abnormality will be suppressed.

Let $N({\bar{x}}_{k}^{i},P_{k}^{i})$ calculated by (20) and (23) be the important density function and conduct importance resampling to obtain the new particle $x_{k}^{i}∼N({\bar{x}}_{k}^{i},P_{k}^{i})$.

**Step 3:** Calculate the weights

(25) $$w_{k}^{i}=w_{k-1}^{i}\frac{p(y_{k}|x_{k}^{i})p(x_{k}^{i}|x_{k-1}^{i})}{q(x_{k}^{i}|x_{k-1}^{i},y_{k})}\;$$

and normalize them as ${\tilde{w}}_{k}^{i}=w_{k}^{i}/\sum_{i=1}^{n}w_{k}^{i}$.

**Step 4:** Calculate the estimate threshold

(26) $${\hat{N}}_{eff}=1/\sum_{i=1}^{N}{\left({\tilde{w}}_{k}^{i}\right)}^{2}\;$$

The severity of particle degeneracy can be determined by comparing the result obtained from (26) with the established threshold. The smaller ${\hat{N}}_{eff}$ is, the worse the particles degeneracy is. In this case, in order to inhibit particles degeneracy, M new particles can be resampled from the posterior density function obtained above. Then, a common weight 1/M is assigned to each new particle.

**Step 5:** Calculate the estimate of the nonlinear state vector

(27) $${\hat{x}}_{k}=\sum_{i=1}^{N}{\tilde{w}}_{k}^{i}{x^{i}}_{k}\;$$

**Step 6:** Go to Step 2 for the state estimation at the next epoch.

In the above recursive process of filtering, the proposed filter constantly checks whether there is a change in the kinematic model. The original kinematic model will be modified according to the change (if any) such that it can adapt to the dynamic change. In other words, the filter itself constantly uses the noise statistical characteristic or gain matrix to reduce the estimated state error, improve the filtering accuracy and provide a better sampling function for the importance sampling process. Simultaneously, the Cholesky factorization of covariance matrices guarantees the stability of the filtering process.

## 4. Performance Evaluation and Discussion

Experimental analysis was conducted to evaluate the performance of the proposed ASUPF. The comparison analysis of ASUPF with EKF, UKF, PF and UPF was also conducted for the performance evaluation.

### 4.1. Experimental Setup

**Figure 1:**
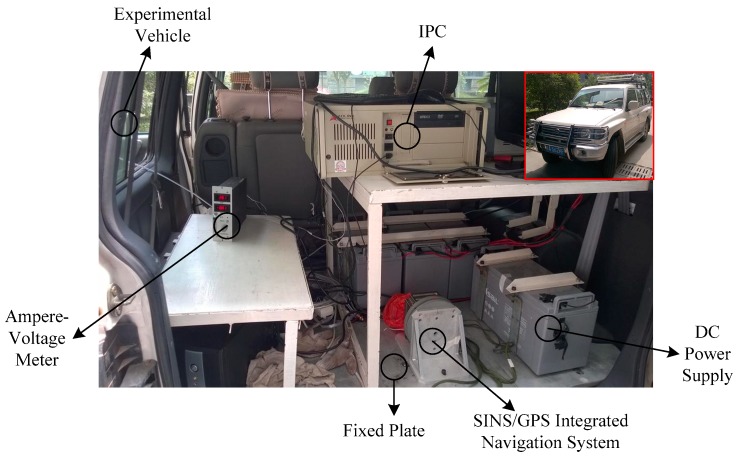
Experimental setup.

An experiment was designed for ground vehicle navigation using a SINS/GPS integrated navigation system. The experimental setup is shown in Figure 1. The test vehicle is a white urban off-road vehicle, where an SINS/GPS integrated navigation system is mounted on the vehicle via the fixed plate dynamic navigation. The vehicle also carries auxiliary facilities including a DC power supply which is mounted on the vehicle via the fixed plate, an industrial personal computer (IPC), a data memory and an ampere-voltage meter. Table 1 provides the specifications of these auxiliary facilities.

**Figure 2:**
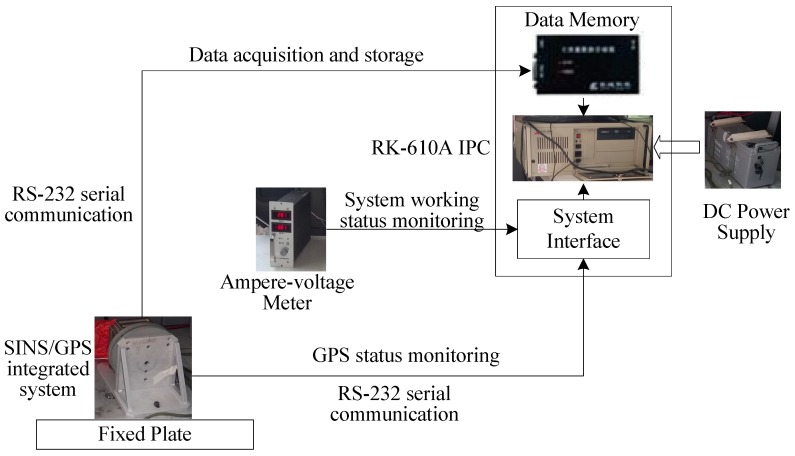
The framework of the experimental system.

The framework of the experimental system is shown in Figure 2. The SINS/GPS integrated navigation system provides SINS measurement, GPS positioning and integrated navigation results (position, velocity and attitude), respectively. These navigation data are stored to the data memory through the RS-232 interface and further transferred to IPC for post-processing and filtering. In addition, the GPS Status Toolbox in IPC is adopted to dynamically monitor the environment for GPS measurement, check the number and distribution of observable GPS constellations and control the GPS initialization and operation. The monitoring data are fed back to IPC through the system interface board. The ampere-voltage meter is used to dynamically measure the current and voltage of the system interface to determine whether the SINS/GPS integrated navigation system works normally or not.

The parameters of the SINS/GPS integrated navigation system are provided in Table 2.

**Figure 3:**
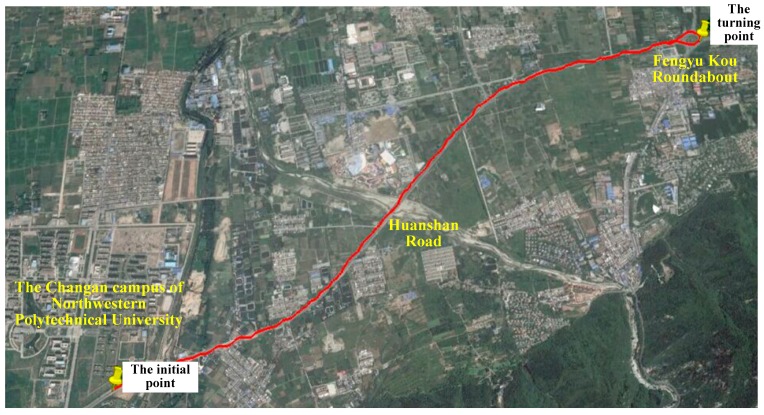
Vehicle traveling trajectory.

**Figure 4:**
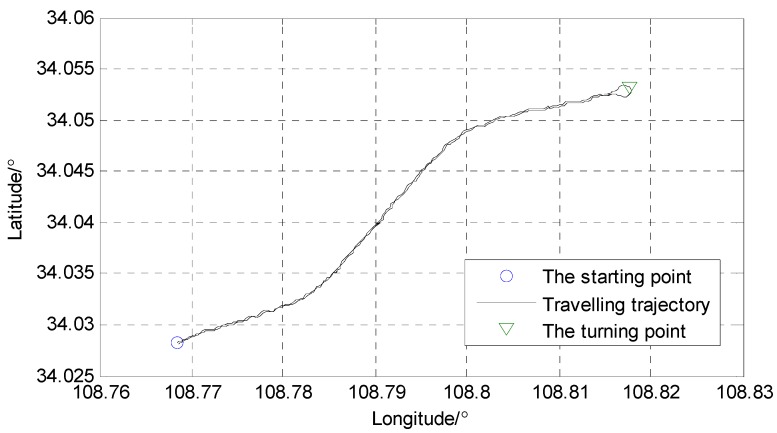
The position coordinates of the vehicle travelling trajectory.

After the one-minute initialization of the SINS/GPS integrated system, the test vehicle started to travel to the East along the Huanshan Road to the Fengyu Kou roundabout. The start position of the vehicle was (E108°46′05.89″, N34°01′41.24″). When arriving at the Fengyu Kou roundabout, the vehicle turned at the position (E108°49′04.61″, N34°03′10.28″) and then travelled back to the start position. The travelling trajectory of the test vehicle and associated position coordinates are shown in Figure 3 and Figure 4, respectively. The travelling distance was 12.38 km, the travelling time was 19 min and the average speed of the vehicle was 39.1 km/h. During the test process, the GPS receiver received signals from at least seven navigation stars. The data obtained from the high-precision differential GPS receiver C-Nav3050 were used as reference for the comparison with the positioning results from the SINS/GPS integration system.

### 4.2. System Models of SINS/GPS Integrated Navigation

The navigation frame is the E-N-U (East-North-Up) geographic coordinate system. The state vector x(t) of the SINS/GPS integrated navigation system is defined as

(28) $$x(t)={\left[\phi_{E},\;\phi_{N},\;\phi_{U},\;\delta v_{E},\;\delta v_{N},\;\delta v_{U},\;\delta L,\;\delta \lambda ,\;\delta h,\;\varepsilon_{x},\;\varepsilon_{y},\;\varepsilon_{z},\;\nabla_{x},\nabla_{y},\;\nabla_{z}\right]}^{\text{T}}$$

where $\left(\phi_{E},\;\phi_{N},\;\phi_{U}\right)$ is the attitude error, $\left(\delta V_{E},\delta V_{N},\delta V_{U}\right)$ is the velocity error, (δL,δλ,δh) is the position error in latitude, longitude and altitude, $\left(\varepsilon_{x},\varepsilon_{y},\varepsilon_{x}\right)$ represents the random drift of the gyroscope and $\left(\nabla_{x},\nabla_{y},\nabla_{z}\right)$ is the constant bias of the accelerometer.

The kinematic model of the SINS/GPS integrated navigation system is expressed as

(29) $$\dot{x}(t)=f(x,t)+G(t)w(t)\;$$

where f(x,t) is the nonlinear state function of the system, $w(t)={\left[w_{gx},w_{gy},w_{gz},w_{ax},w_{ay},w_{az}\right]}^{T}$ is the system noise consisting of gyro’s Gaussian white noise $\left(w_{gx},w_{gy},w_{gz}\right)$ and accelerometer’s Gaussian white noise $\left(w_{ax},w_{ay},w_{az}\right)$ and G(t) is the coefficient matrix of the system noise.

The observation model is described as

(30) $$z_{k}=\delta \rho_{k}=H_{k}x_{k}+\frac{1}{2}\left[\begin{array}{c} x_{k}^{T}\cdot \left(D_{v}^{T}\cdot {C_{g}^{e}}^{T}\cdot H^{\left(1\right)}\left(r_{ins}\right)\cdot C_{g}^{e}\cdot D_{v}\right)\cdot x_{k}\\ x_{k}^{T}\cdot \left(D_{v}^{T}\cdot {C_{g}^{e}}^{T}\cdot H^{\left(2\right)}\left(r_{ins}\right)\cdot C_{g}^{e}\cdot D_{v}\right)\cdot x_{k}\\ x_{k}^{T}\cdot \left(D_{v}^{T}\cdot {C_{g}^{e}}^{T}\cdot H^{\left(3\right)}\left(r_{ins}\right)\cdot C_{g}^{e}\cdot D_{v}\right)\cdot x_{k}\\ x_{k}^{T}\cdot \left(D_{v}^{T}\cdot {C_{g}^{e}}^{T}\cdot H^{\left(4\right)}\left(r_{ins}\right)\cdot C_{g}^{e}\cdot D_{v}\right)\cdot x_{k} \end{array}\right]+v_{k}\;$$

where $\delta \rho_{k}$ is the pseudo-range difference of GPS satellites, $H_{k}$ is the observation matrix, $v_{k}$ is the observation noise, $C_{g}^{e}$ is the transformation matrix from the geographic coordinate system to the earth coordinate system, $r_{ins}$ is the INS position vector and $D_{v}$ is an auxiliary matrix, which is expressed as

(31) $$D_{v}=\left[\begin{array}{ccc} 0_{3\times 6} & I_{3\times 3} & 0_{3\times 8} \end{array}\right]\;$$

### 4.3. Filtering Accuracy

For comparison analysis, trials based on the above experimental design were conducted by using EKF, UKF, PF, UPF and ASUPF, respectively. The unscented transformation parameters were α=0.5 and β=2. The adaptive factor calculation parameters were $c_{0}=1$ and $c_{1}=3.5$. The sampling time was 1000 s. 50 Monte Carlo simulations were conducted for each of the five filters.

**Figure 5:**
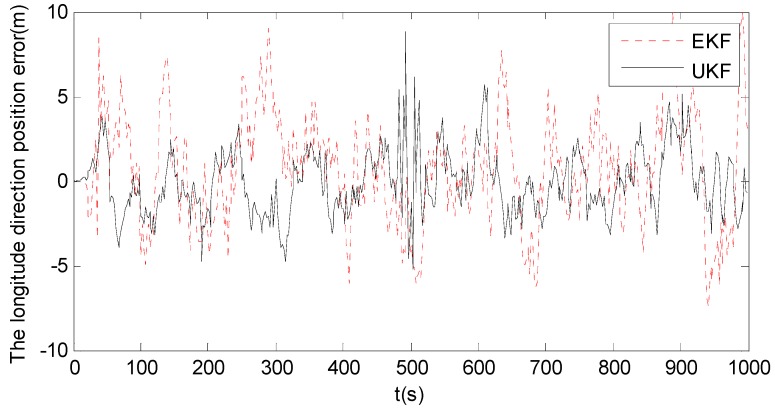
The longitude errors of EKF and UKF.

Since the position errors in the other directions have the similar trends as that in the longitude direction, only the position error in the longitude direction is discussed for conciseness. Figure 5 shows the longitude errors of EKF and UKF. It can be seen that EKF has the poor filtering accuracy, due to the error caused by the linearization of the nonlinear state model. Although UKF improves the filtering accuracy of EKF, the improved accuracy is still limited. This is because UKF approximates the posterior probability distribution of the system state using the Gaussian distribution. Its filtering accuracy is significantly degraded when the posterior probability distribution of the system state is non-Gaussian distribution, which is the case of the experimental test. Therefore, both EKF and UKF have limited accuracy for strongly nonlinear systems.

**Figure 6:**
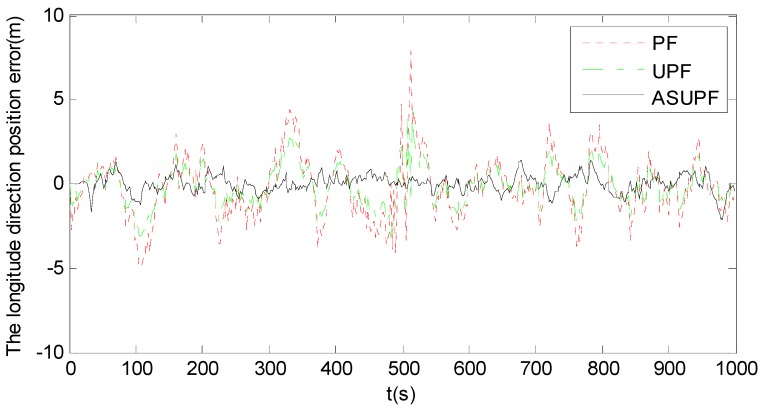
The longitude errors of PF, UPF and ASUPF (M=200).

Figure 6 shows the longitude errors of PF, UPF and ASUPF, where the particle number is M=200. Compared to Figure 5, it is obvious that all three particle filters (PF, UPF and ASUPF) have higher accuracy than both EKF and UKF. This is mainly because these three particle filters describe the priori and posteriori information using samples instead of a function, thus overcoming the limitation of both EKF and UKF that random variables must satisfy the Gaussian distribution. However, PF suffers from the particle degradation phenomenon, leading to the limited filtering accuracy. UPF improves the filtering accuracy of PF, as it generates the importance function and conducts resampling using UT to weaken the phenomenon of particle degradation. However, due to the influence of abnormal interference on the state estimation, the filtering curve of UPF still involves large oscillations. As clearly shown in Figure 6, the abnormal interference caused by the sharp U-turn travelling at around t=500 s significantly affects the performances of PF and UPF. In contrast, ASUPF improves UPF by introducing the adaptive factor to suppress the influence of abnormal interference on the kinematic and observation models. Therefore, ASUPF has much higher accuracy than both PF and UPF. Table 3 lists the root mean square errors (RMSEs) in the longitude direction for each nonlinear filter.

**Figure 7:**
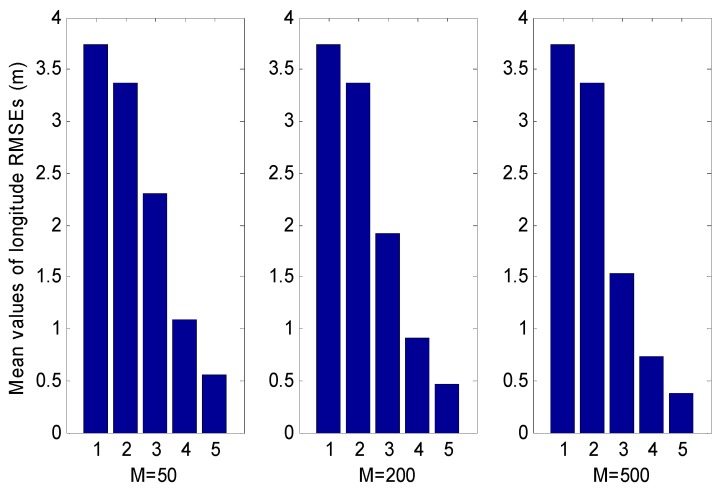
Mean values of the longitude RMSEs of EKF, UKF, PF, UPF and ASUPF, where the mean values for PF, UPF and ASUPF are subject to different particle numbers M=50, M=200 and M=500 and the numbers from 1 to 5 indicate EKF, UKF, PF, UPF and ASUPF, respectively.

Figure 7 shows the means of the longitude RMSEs for the five filters, where the means of the RMSEs of the three particle filters (PF, UPF and RAUPF) are subject to three different particle numbers M=50, M=200 and M=500. It can be seen that both EKF and UKF involves a large error. However, all three particle filters still have higher accuracy than both EKF and UKF, even with the small number of particles (M=50).

### 4.4. Computational Performance and Filtering Robustness

Trials were conducted with Matlab programs on a 2.93 GHz dual-core CPU and 2 G RAM PC to analyse the computational performances of EKF, UKF, PF, UPF and ASUPF, where the particle number was set to M=200 for PF, UPF and ASUPF. Table 4 shows the computational performances of each filter.

It can be seen that the computational times of PF, UPF and ASUPF are obviously larger than those of EKF and UKF. This is because the computational processes of these three particle filters are more complex, involving sampling a large number of particles, allocating weights and resampling. Thus, they require more CPU utilizations.

In order to analyse the robust performances of EKF, UKF, PF, UPF and ASUPF, the above experimental data were divided into two groups. One was within the sharp U-turn time period (484.2 s, 512.8 s) and the other was within the rest time periods. Based on each group of experimental data, the longitude RMSEs of EKF, UKF, PF, UPF and ASUPF were calculated, where the particle number was set to M=200 for PF, UPF and ASUPF. The RMSE differences between the two groups of experimental data indicate the robust performances of each filter. Table 5 shows the results on the robustness of each filter.

It can be seen from Table 5 that abnormal disturbances affect EKF, UKF and PF more significantly than UPF and ASUPF. This is also in agreement with the oscillations in the error curves of EKF, UKF and PF as shown in Figure 5 and Figure 6. However, the influence of abnormal disturbances on ASUPF is even more than twice smaller than that on UPF. This is because ASUPF can control the noise statistics of the kinematic and observation models by adjusting the adaptive factor to suppress the influence of abnormal interferences.

### 4.5. Overall Performance

Define the overall performance index of a filtering algorithm as

(32) $$S=f(p,R_{T},R)=\frac{1}{W\cdot {\left(p,R_{T},R\right)}^{T}}\;$$

where S represents the overall performance index of the filtering algorithm and the larger the value is, the better the performance of the algorithm is p, $R_{T}$ and R are the three performances of the filtering algorithm, that is, the accuracy, computational performance and robustness, respectively. $W=(\beta_{1},\beta_{2},\beta_{3})$, where $\beta_{i}$ (i=1,2,3) are the weights of the three performances, respectively, and

(33) $$\sum_{i=1}^{3}\beta_{i}=1\;\begin{array}{c} 0\leq \end{array}\beta_{i}\leq 1$$

Under different performance requirements of a navigation system, the value of $W=(\beta_{1},\beta_{2},\beta_{3})$ is different. For example, W=(0.6,0.2,0.2) indicates that the priority of the navigation system is the positioning accuracy, while the computational performance and robustness are subservient to the positioning accuracy.

Table 6 shows the overall performance indices of EKF, UKF, UPF and ASUPF under three different priorities of accuracy, computational performance and robustness (represented by the three values of **W**), where the values of p, $R_{T}$ and R correspond to the normalized values of the three performances as shown in Table 3, Table 4 and Table 5, respectively.

The overall performance indices of EKF and UKF under the three different priorities show that both EKF and UKF have a strong advantage in the computational performance. Although both accuracy and robustness are weak, the accuracy performance is better than the robustness performance for both EKF and UKF. This is also in agreement with the experimental results of EKF and UKF (see Figure 5 and Figure 7 and Table 5).

The overall performance indices of ASUPF under the three different priorities show that ASUPF has strong accuracy and robustness performances and its robustness is highest in Table 6. This proves that the improvement of ASUPF in adaptability and stability is effective. Although the computational performance of ASUPF is lower than those of EKF and UKF, ASUPF has a much better overall performance than the other filters for the integrated navigation system.

In general, the performance requirements of a navigation system determine the selection of an appropriate filter. For a navigation system desiring high accuracy and strong robustness, ASUPF should be considered. For a navigation system desiring a high computational performance, either EKF or UKF should be considered.

## 5. Conclusions

This paper presents a new ASUPF for nonlinear systems by combining adaptive filtering and square-root filtering into UPF. This algorithm improves UPF by using the adaptive factor to refrain from the disturbances of the noise statistics of observation and kinematic models, thus overcoming the particle degeneracy problem involved in UPF. It also applies Cholesky factorization to suppress the negative definiteness of the covariance matrices of predicted state vector and observation vector. Experiments and comparison analysis demonstrate that the proposed ASUPF can effectively prevent particles from degeneracy and improve the filtering accuracy of dynamic navigation. Future work will focus on the sensitivity analysis of the proposed ASUPF in comparison with the existing nonlinear filtering algorithms such as EKF, UKF, PF and UPF.

## Figures and Tables

**Table 1:** Specifications of the auxiliary facilities.

Item	Model	Specifications
IPC	ADLINK RK-610A	It is a 2.73 GHz Intel Core Duo CPU and 2.00 GB RAM PC, installed with GPS Status Toolbox PRO v5.1. This PC is equipped with a navigation system interface board and a 17-inch LCD monitor.
Data memory	LCW-S02	It has a RS-232/485 interface, the storage rate is 10 KB/s and the storage capacity is 32 G that can be expanded. The optional baud rate is 4800~115,200 bps. The file system is FAT32 and the storage file format is * .txt. The operating temperature is −35 °C~85 °C.
DC power supply	Sail 6-GFM-100	It consists of four groups of sustainable and stable discharge batteries, where each battery rated voltage is 12 V and the rated capacity is 30.0 AH (10 h and the termination voltage of 10.8 V).
Ampere-voltage meter	Transmit G-2505	The voltage range is 0~50 V, the current range is 0~5 A and the measurement accuracy is 0.5% FS.
Fixed plate	—	It is a 10 mm thick steel plate with screw holes and bracket.

**Table 2:** The parameters of the SINS/GPS integrated navigation system.

Parameter		Value
Update Rate		SINS 125 Hz, GPS 5 Hz
Start Time		<1 s
Operating Temperature		−30 °C~+60 °C
Angular Velocity Measurement	Measuring Range	±200 °/s
	Zero-bias Stability	10.0 °/h (1σ)
	Scale Factor	0.1% (1 σ)
	Non-linear	0.01% FS (1σ)
	Random Walk Coefficient	1.0 °/hr^1/2^ (1σ)
Acceleration Measurement	Measuring Range	±20 g
	Zero-bias Stability	2 mg (1σ)
	Scale Factor	0.1% (1σ)
	Non-linear	0.01% FS (1σ)
	Random Walk Coefficient	0.005 m/s/hr^1/2^ (1σ)
GPS Measurement	L1/L2	Horizontal Accuracy 1.0 m, Vertical Accuracy 1.5 m (1σ)
	SBAS	Horizontal Accuracy 0.6 m, Vertical Accuracy 1.0 m (1σ)
	DGPS	Horizontal Accuracy 0.3 m, Vertical Accuracy 0.5 m (1σ)
	Velocity Accuracy	0.02 m/s (1σ)

**Table 3:** The mean values of the longitude RMSEs for EKF, UKF, UPF and ASUPF.

Filter	Mean Value of Longitude RMSEs/m	Normalized Mean Value
EKF	3.62	0.7240
UKF	2.56	0.5120
PF (M=200)	2.13	0.4260
UPF (M=200)	1.15	0.2300
ASUPF (M=200)	0.46	0.0920

**Table 4:** Computational performances of EKF, UKF, PF, UPF and ASUPF.

Filter	Equivalent Computational Complexity	Peak of CPU Utilization	Running Time/s	Normalized Running Time/s
EKF	$O(n^{3})$	18%	0.202	0.0505
UKF	$O(n^{4})$	23%	0.958	0.2395
PF	$O(Mn^{3})$	42%	2.411	0.6028
UPF	$O(Mn^{3}+n^{4})$	48%	3.078	0.7695
ASUPF	$O(Mn^{3}+n^{4})$	49%	3.089	0.7722

**Table 5:** Robust performances of EKF, UKF, PF, UPF and ASUPF.

Filter	Longitude Direction Position RMSE/m			Normalized Difference
	The Sharp U-turn Time Period	The Other Time Periods	Difference	
EKF	5.3584	3.5753	1.7831	0.8915
UKF	4.1364	2.8243	1.3121	0.6561
PF	3.1658	2.0370	1.1288	0.5644
UPF	1.5469	0.8663	0.6806	0.3403
ASUPF	0.5517	0.4191	0.1326	0.0663

**Table 6:** Overall performance indexes of the nonlinear filtering algorithms.

Filtering Algorithms	Accuracy Priority W=(0.6,0.2,0.2)	Timing Priority W=(0.2,0.6,0.2)	Robustness Priority W=(0.2,0.2,0.6)
EKF	1.6056	2.8296	1.4496
UKF	2.0563	2.6503	1.8385
PF	2.0449	1.7866	1.8369
UPF	2.7781	1.7368	2.4748
ASUPF	4.4861	2.0202	4.7030
